# Sensorimotor performance in acute-subacute non-specific neck pain: a non-randomized prospective clinical trial with intervention

**DOI:** 10.1186/s12891-021-04876-4

**Published:** 2021-12-04

**Authors:** Renaud Hage, Christine Detrembleur, Frédéric Dierick, Jean-Michel Brismée, Nathalie Roussel, Laurent Pitance

**Affiliations:** 1 Laboratoire de Neuro Musculo Squelettique (NMSK), Institut de Recherche Expérimentale et Clinique (IREC), Université catholique de Louvain, Avenue Mounier 53, B1.53.07 –, 1200 Brussels, Belgium; 2 Centre de Recherche et de Formation (CeREF), HELHa, Mons, Belgium; 3 Traitement Formation Thérapie Manuelle (TFTM), Private Physiotherapy Center, Brussels, Belgium; 4 Laboratoire d’Analyse du Mouvement et de la Posture (LAMP), Centre National de Rééducation Fonctionnelle et de Réadaptation (Rehazenter), Luxembourg, Luxembourg; 5 Center for Rehabilitation Research, Texas Tech University Health Sciences Center, Lubbock, TX USA; 6 Rehabilitation Sciences and Physiotherapy (MOVANT), Faculty of Medicine and Health Sciences, University of Antwerp, Antwerp, Belgium; 7 Stomatologie et Chirurgie Maxillo-Faciale, Cliniques Universitaires Saint-Luc, Brussels, Belgium

**Keywords:** Cervical rotation, sensorimotor assessment, acute non-specific neck pain, passive mobilizations

## Abstract

**Background:**

The assessment of cervical spine kinematic axial rotation performance is of great importance in the context of the study of neck sensorimotor control. However, studies addressing the influence of the level of provocation of spinal pain and the potential benefit of passive manual therapy mobilizations in patients with acute-subacute non-specific neck pain are lacking.

**Methods:**

A non-randomized prospective clinical trial *with* an intervention design was conducted. We investigated: (1) the test-retest reliability of kinematic variables during a fast axial head rotation task standardized with the DidRen laser test device in 42 Healthy pain-free Control Participants (HCP) (24.3 years ±6.8); (2) the differences in kinematic variables between HCP and 38 patients with Acute-subacute Non-Specific neck Pain (ANSP) assigned to two different groups according to whether their pain was localized in the upper or lower spine (46.2 years ±16.3); and (3) the effect of passive manual therapy mobilizations on kinematic variables of the neck during fast axial head rotation.

**Results:**

(1) Intra-class correlation coefficients ranged from moderate (0.57 (0.06-0.80)) to excellent (0.96 (0.91-0.98)). (2) Kinematic performance during fast axial rotations of the head was significantly altered in ANSP compared to HCP (age-adjusted) for one variable: the time between peaks of acceleration and deceleration (*p*<0.019). No significant difference was observed between ANSP with upper *vs* lower spinal pain localization. (3) After the intervention, there was a significant effect on several kinematic variables, e.g., ANSP improved peak speed (*p*<0.007) and performance of the DidRen laser test (*p*<0.001), with effect sizes ranging from small to medium.

**Conclusion:**

(1) The DidRen laser test is reliable. (2) A significant reduction in time between acceleration and deceleration peaks was observed in ANSP compared to HCP, but with no significant effect of spinal pain location on kinematic variables was found. (3) We found that neck pain decreased after passive manual therapy mobilizations with improvements of several kinematic variables.

**Trial registration:**

Registration Number: NCT 04407637

**Supplementary Information:**

The online version contains supplementary material available at 10.1186/s12891-021-04876-4.

## Introduction

Approximately 50% of the population suffers from neck pain at least once in their lifetime, with women being more at risk than men [1]. Neck pain results in high healthcare costs and is the fourth leading cause of disability [2], ranking second only to low back pain in selected countries [3].

Most patients with neck pain are nowadays classified as suffering from “non-specific” neck disorder [4–6]. Since the main purpose of a diagnosis and a classification system is to make predictions and provide the best therapeutic approach [7], this classification does not help clinicians in their clinical reasoning to understand the factors contributing to the patient’s pain and dysfunction.

“Non-specific” refers to pain in the neck that occurs without trauma, signs or symptoms of major structural pathology, neurological signs, or specific pathology [4]. Degenerative musculoskeletal changes and/or psychosocial stress can alter the somatosensory inputs of the cervical spine in many patients with non-specific neck pain, resulting in functional changes such as lack of stability and impaired kinematic control [8–10].

Patients with non-specific neck pain therefore present specific issues for clinicians who must treat the patients’ pain. Classifying patients with neck pain into specific and non-specific categories is certainly a first step in the process of clinical reasoning. But this classification alone does not allow a complete treatment plan to be established. Therefore, a history and a thorough clinical examination are important to guide the clinical reasoning process. The clinical guidelines for the management of patients with neck pain recommended by the Orthopedic Section of the American Physical Therapy Association (APTA) use a classification system based on the International Classification of Function impairments (ICF) for body functions terminology [4]. They recommend including assessment of range of motion and response to pain [11, 12]. However, to obtain a more complete clinical picture of patients with neck pain during movements, other objective observations such as the quality of movement (i.e., sensorimotor appraisal) are essential.

Clinicians are showing increasing interest in various tests to better define the clinical picture of patients by focusing on the assessment of sensorimotor control during axial head rotation [13–19]. Calculating the error in repositioning the head, measuring accuracy in tracking a virtual target, or assessing accurate fast axial rotation of the head in response to real visual targets are all possible assessments of cervical spine sensorimotor control [18, 20, 21]. The DidRen laser is a functional test consisting of standardized task in which axial head rotations are performed from “target-to-target” in the same sequence. It consists of fast, precise, low-amplitude axial rotations of the head in response to real visual targets that must be hit by a laser beam placed on the subject’s head [21–23]. This test is particularly useful because it focuses on the sensory and motor control systems of the neck and has many direct neurophysiological connections between the proprioceptive, visual, and vestibular systems [8]. Head rotation requires special attention because axial rotation of the head is one of the most frequently performed movements of the neck during activities of daily living [24]. Moreover, a small amount of head rotation (<30°) seems to correspond most closely to the normal functional range of motion of the cervical spine during activities of daily living (i.e. ± 20°) [25]. Furthermore, limiting the head rotation to 30° [26] avoids stressing the passive cervical spine system (joint capsules, facet joints, intervertebral disks, and ligaments) and focuses on input from the proprioceptive system of the upper cervical spine, which is highly developed in the sub-occipital upper neck region [27, 28] and corresponds to the spinal muscles that provide dynamic stability during the first degrees of rotation [26].

Most studies of sensorimotor control have been conducted in patients suffering from chronic neck pain [29–31]. Nevertheless, there is some evidence that sensorimotor control deficits can occur shortly after the onset of neck pain [29], as demonstrated in patients after acute whiplash trauma [29]. However, there is a lack of studies in patients with acute-subacute non-specific neck pain. We do not yet know whether the assessment of movement quality (i.e., in terms of kinematic strategies) assessed with an axial rotation test is reliable in patients with acute-subacute non-specific neck pain and whether differences in kinematics are observed compared with healthy controls. In addition, the localization of the pain may play a role. Although there is some evidence that sensorimotor dysfunction is more important in participants with chronic neck pain originating from upper cervical levels (C_0_ to C_2_) than from the lower cervical levels (C_3_ to C_7_) [30, 31], to our knowledge, there are no studies examining differences in sensorimotor dysfunction based on the level of pain provocation in the spine.

Finally, there are a growing number of studies demonstrating the effects of passive spinal manipulations on sensory processing, motor performance, functional performance, sensorimotor integration [32, 33], and pain relief [34, 35]. However, studies investigating the potential benefits of passive manual mobilization on changes in sensorimotor control in patients with acute-subacute non-specific neck pain are lacking.

Therefore, this study examined sensorimotor performance during the DidRen laser test [36]. We examined the test-retest reliability of kinematic neck rotation variables in Healthy pain-free Control Participants (HCP) (i.e. Aim 1) [23]; the differences in kinematic variables between HCP and patients with Acute-subacute Non-Specific neck Pain (ANSP) (i.e. Aim 2); and the effect of passive manual therapy mobilizations on neck kinematic variables (i.e. Aim3). We hypothesized that the test-retest reliability of neck kinematic rotation variables would be acceptable (Aim 1), that the kinematic variables of ANSP patients and particularly those suffering from upper neck pain would be significantly impaired compared to HCP (Aim 2), and that neck kinematic rotation variables would improve after pain relief from passive manual therapy mobilizations (Aim 3*)* [37–40].

## Methods

### Study design

**Fig. 1:**
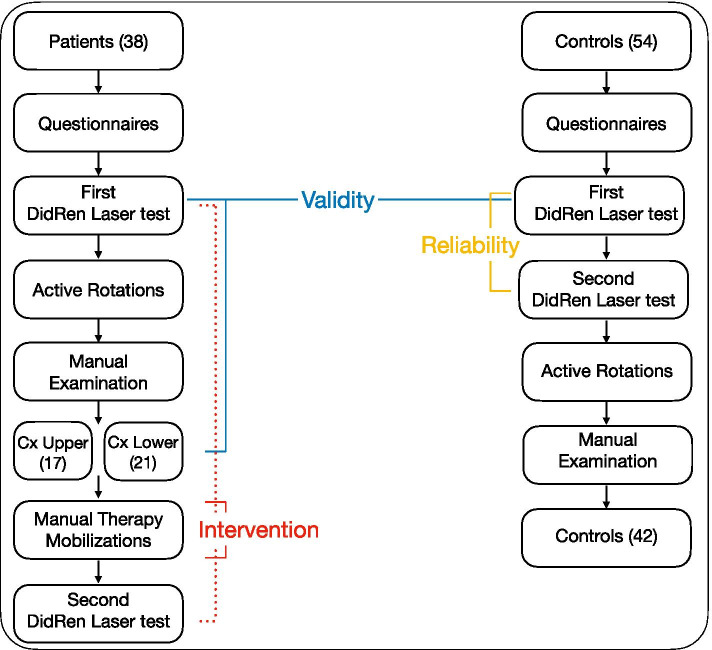
Flow chart. Inclusion of 38 Patients (ANSP) and 42 Controls (HCP) with the three aims of the study presented: Reliability (test-retest reliability of kinematic variables), Validity (differences in kinematic variables between HCP and ANSP (upper *vs* lower spine pain localization: based on the manual examination, 38 ANSP were assigned to either the upper (C_0_-C_2_; *n*=17) or lower (C_3_ to C_7_; *n*=21) spine pain group)), and Intervention (effect of manual therapeutic interventions on kinematic variables assessed between the first and second DidRen laser tests). For blinding reasons, a first examiner supervised the completion of the questionnaires, the DidRen laser test, and the active cervical spine rotation test (range of motion). The questionnaires included the Neck Disability Index (NDI), the French version of the Bournemouth questionnaire (BQ), the Tampa scale of Kinesiophobia (TSK) (except for HCP), and the Numeric Pain Rating Scale (NPRS). *A second examiner performed the cervical spine manual examination and the passive manual therapy mobilization sessions*. Based on the manual examination, 12 HCP who experienced pain were excluded from the study; 42 HCP were then included

The present study entailed a non-randomized prospective clinical trial *with* intervention (Fig. 1). The protocol allowed us to investigate the reliability, validity, and interventional part of our experimentation.

### Participants

A consecutive sample of patients diagnosed as ANSP by general practitioners was recruited from February to December 2019 in a private manual physiotherapy center in Brussels (Belgium). Inclusion criteria were acute-subacute (<3months) non-specific neck pain with a Neck Disability Index (NDI) > 8% [41] and a Numeric Pain Rating Scale (NPRS) >3 [33, 42–45]. ANSP were excluded if they reported any of the following: a history of neck surgery, dizziness due to neck or head movements [46], and physician-diagnosed cervical radiculopathy [47], as these could affect neck sensorimotor control. HCP were recruited from a sample of convenience from colleagues at the university hospital and from the researchers’ acquaintances. They were included if they reported no neck symptoms: i.e. the NDI < 8% [41] and an NPRS =0 [42]. HCP were excluded if they reported neck pain, radiating shoulder or upper limb symptoms, or headache in the past year, or if they had a history of neck trauma or were receiving treatment for spinal disorders (conservative treatment/surgery) [46]. HCP were also excluded if they reported dizziness or pain with active head rotation or manual spinal assessment [48].

All participants signed an informed consent form, and the study was approved by the Comité Académique de Bioéthique (https://www.a-e-c.eu, Brussels, B200-2018-103) and conducted in accordance with the Declaration of Helsinki. The authors confirm that all ongoing and related trials for this drug/intervention are registered (ClinicalTrials.gov: 04407637).

### Questionnaires

At baseline, patients were asked to fill in the following questionnaires: the French version of the NDI, the French version of the Bournemouth questionnaire (BQ) [49], the French version of the Tampa scale of Kinesiophobia (TSK) [50] and the NPRS. HCP were asked to complete the NDI, the BQ and the NPRS.

The NDI is a self-rated questionnaire assessing disability due to neck pain, consisting of a series of 10 questions about activities of daily living, all scored on a 6-point scale. Each item is scored at 5 points, resulting in a maximum total score of 50 or a percentage of 100. The NDI score (in %) is interpreted as follows: 0-8 = none; 10-28 = mild; 30-48 = moderate; 50-68 = severe; more than 68 = complete [51]. The NDI has shown good to excellent clinometric properties in patients with neck pain [41, 51, 52].

The BQ evaluates several dimensions participants with neck pain, including pain, disability, affective and cognitive aspects of neck pain. Each question (7 items) is scored on an eleven-point (0-10) numeric rating scale. The maximum score for the BQ is 70 points and is the sum of the scores for each of the seven items [49]. The BQ has shown good to excellent clinometric properties in patients with neck pain [49].

The TSK is a 17-item questionnaire assessing fear of movement or reinjury, in which participants are asked to rate their level of agreement with each item on a scale of 1 (strongly disagree) to 4 (strongly agree). The TSK has been shown to be associated with measures of behavioral avoidance and self-reported disability. A cut-off score of 39 is associated with risk for prolonged pain-related disability [53]. The TSK has demonstrated moderate clinometric properties in patients with neck pain [53].

The NPRS is commonly used to assess patients with neck pain. It uses an 11-point scale ranging from 0 (no pain) to 10 (worst pain imaginable) [42]. The NPRS has shown excellent clinometric properties in patients with neck pain [54, 55].

After completing the questionnaires, ANSP were assigned to the examination phases: DidRen laser test and manual examination. The manual examination was used to assign the ANSP to the group with the upper or lower cervical origin. Then, both groups of ANSP underwent passive manual mobilization sessions. After the last session, they were immediately examined for the DidRen laser test, which allowed us to calculate the effect of the intervention on the neck rotation kinematic variables.

After completing the questionnaires, the HCP repeated the DidRen laser test a second time. In this way, we were able to calculate the reliability of the neck rotation kinematic variables. After the second test, HCP underwent a manual examination. HCP who were symptomatic on manual examination were removed from the study as they were no longer considered “pain-free healthy controls”. Comparing the results of the DidRen laser test of ANSP and the remaining healthy control participants allowed us to calculate the validity of kinematic neck rotation variables.

### Examination phases

For blinding, a first examiner supervised the completion of the questionnaires, the DidRen laser test, and the active cervical rotation test (range of motion). A second examiner (RH), who was blinded to the results of the first examiner’s DidRen laser test, performed the cervical manual examination and passive manual mobilization sessions. RH has 20 years of experience as a certified orthopedic manual physical therapist and 15 years of experience as an orthopedic manual therapy instructor.

### DidRen laser test and calculated kinematic variables

**Fig. 2:**
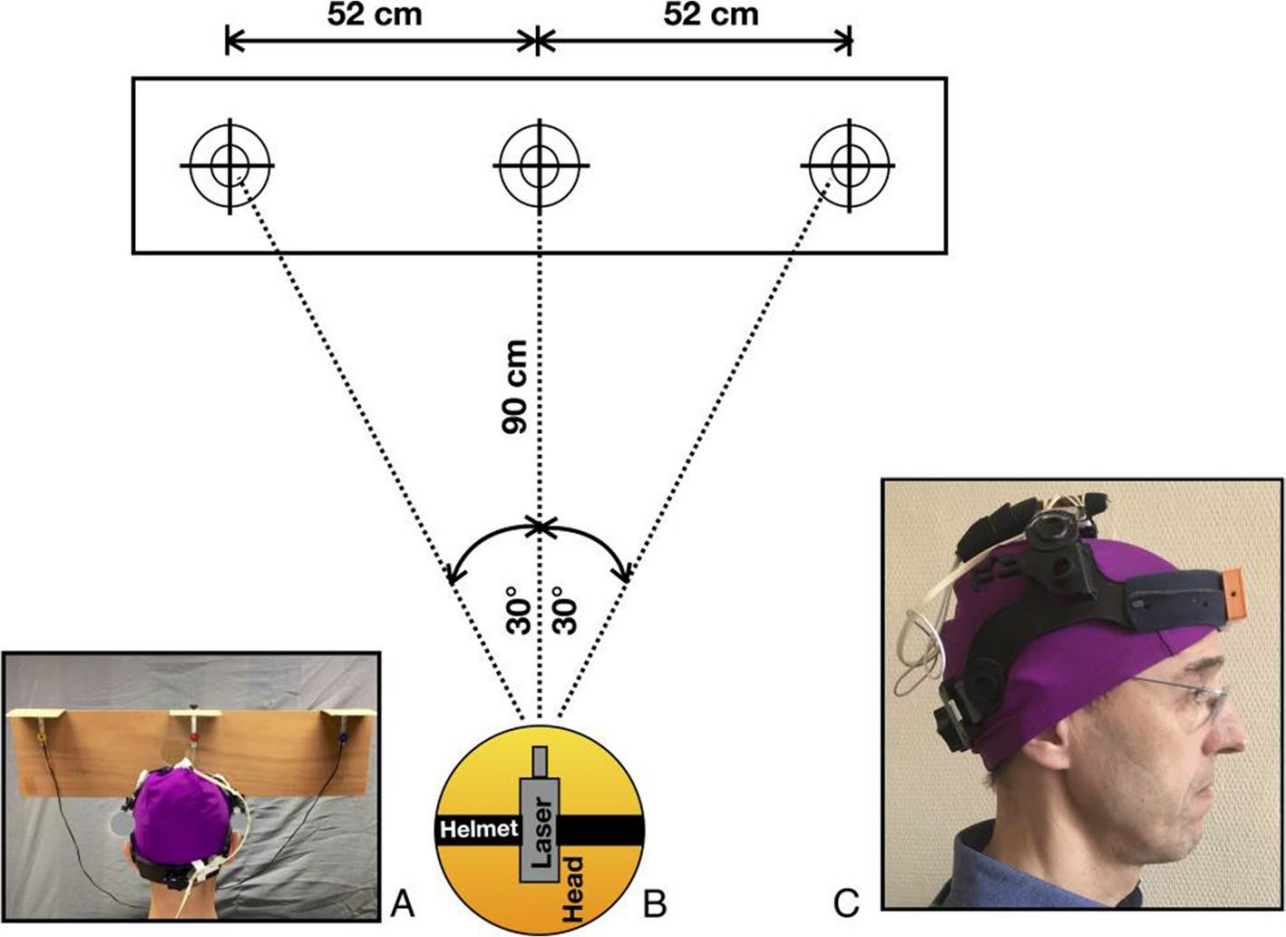
Installation of the DidRen laser test. **A** Head position in front of targets. **B** Schematic top view of the test setup with the three photosensitive sensors. **C** Helmet worn by participant with laser on top and DYSKIMOT on forehead

The DidRen laser test was used to homogenize the head-neck complex rotational motion of the participants as described in previous publications [21–23]. Briefly, participants wore a helmet to which a laser was attached. They directed the laser as fast as possible on three targets equipped with photosensitive sensors (Fig. 2A). To achieve a maximum head rotation of 30°, the sensors were spaced apart and placed at a distance of 90 cm in front of them (Fig. 2B). One test consisted of 5 cycles of right/left rotations.

During the DidRen laser test, axial head rotation was recorded using a validated inertial motion unit sensor, the DYSKIMOT [36]. The DYSKIMOT, which was attached to the front of the helmet, recorded the angular displacement of the head in 3D at a sampling frequency of 100 Hz (Fig. 2C). The homemade DidRen software calculated the time required for the participant to move from one “hit” sensor to the next, and the DidRen total time (in s) to complete the 5 cycles of a trial [21].

**Fig. 3:**
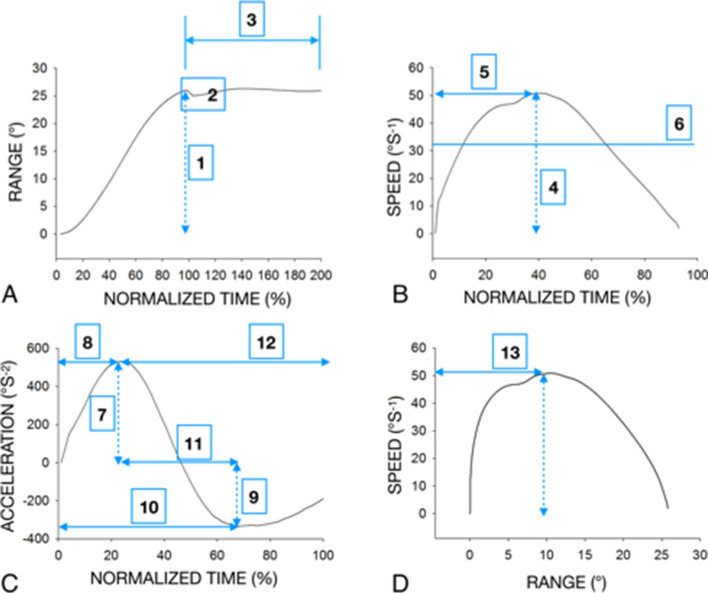
Typical plots of variables analyzed during a right rotation in a HCP from the younger adult group (age: 22 yrs., sex: female). We calculated the angular speed and acceleration of the head-neck complex from the beginning to the end of each rotation cycle. All values are expressed in absolute values. **A** (1) range of motion during the test (ROM test in °); (2) overshoot (°s^-1^); (3) stabilization time (s); (4) peak speed (°s^-1^); **B** (5) time to peak speed (s); (6) average speed (° s^-1^); (7) peak acceleration (°s^-2^); **C** (8) time to peak acceleration (s); (9) peak deceleration (°s^-2^); (10) time to peak deceleration (s); (11) time between peaks of acceleration and deceleration (s); (12) time from peak acceleration to end of rotation (s); **D** (13) angle at maximum speed (°)

As described in Hage et al. (2019), 14 kinematic parameters were calculated to assess the reliability and validity parts. Thirteen specific kinematic parameters from each angular displacement (Fig. 3) and one from the DidRen software. All variables were calculated and averaged during 5 consecutive cycles.

To determine the number of test repetitions required to familiarize the patient/healthy control with the test, we conducted a pilot study with 7 healthy subjects. This pilot study showed us that the DidRen Laser test needed to be performed 4 times. Then we recorded the fourth test outcome for the results.

### Active cervical rotation range of motion (ROM)

The mean value of three active ROM was measured using the DYSKIMOT device [36]: participants were asked to rotate their head and neck as far as possible. During active cervical ROM measurements, participants were asked to indicate any familiar pain on an NPRS (0-10).

### Manual examination of the cervical spine

Based on the manual examination, ANSP were assigned to either the upper (C_0_-C_2_) or lower (C_3_ to C_7_) spinal pain group.

Based on the manual examination, HCP were excluded from the study if they had any symptoms as they were no longer considered “healthy”.

The manual examination of the spine included the C_0_-C_2_ axial rotation test (see Supplementary Fig. 1) [52–54], the passive physiological intervertebral movements (PPIVM’s) (see Supplementary Fig. 2) and the passive accessory intervertebral movements (PAIVM’s) (see Supplementary Fig. 3) [47, 56]. The aim of these tests was to reproduce the patient’s familiar pain. In these tests, the patient was pragmatically asked to indicate their *familiar pain* provocation ≥3 on the NPRS (1-10) [48, 57] when the examiner perceived resistance (subjectively recorded as mild, moderate, marked) [58]. An ANSP patient was classified as “upper spine group” if the patient recognized her/his familiar pain at levels C_0_-C_2_ when the examiner assessed stiffness with the axial rotation test C_0_-C_2_ and/or the PPIVM’s and PAIVM’s at levels C_0_-C_1_-C_2_. An ANSP patient was classified as “lower spine group” if the patient recognized her/his familiar pain below C_2_ when the examiner assessed stiffness below C_2_ with PPIVM’s and PAIVM’s. If the patient reported pain at more than one level of the cervical spine (upper/lower), the examiner selected only the level that reflected the patient’s familiar pain.

Because manual examination of the pain for segmental tenderness is known to have high sensitivity (92%), HCP were excluded if they had pain at one or more cervical spine levels [48].

## Reliability

Once the questionnaires were completed, HCP were assigned to examination part. The reliability of the DidRen laser test and active cervical rotation range of motion was assessed for HCP using the intra-class correlation coefficient (ICC), the standard error of measurements, and the minimum detectable changes.

### Validity

After completing the questionnaires, ANSP were assigned to the spinal region examination part, which included assessment of fast neck rotation with the DidRen laser test, active cervical rotation range of motion, and manual examination. Then the patient was assigned to the intervention phase. Note that the DidRen laser test was assessed by a different examiner to ensure blinding.

### Intervention

The intervention for the patient (i.e. physiotherapy treatment) included PAIVM’s mobilizations [37, 40]. As there is already evidence that motor functions are altered by specific modes of training [59], we tried to avoid and prevent direct interference with the sensorimotor system and the DidRen laser test during intervention phase. Therefore, neither cervical muscle strength-endurance nor functional strength [60] nor sensorimotor control tests (e.g. cervical repositioning, oculomotor exercises [8]) nor head rotation were carried out during the intervention. Pragmatically, PAIVM’s were mainly performed at the spinal level(s) recorded as familiar painful sites during the first spinal examination, but also (if necessary) at other spinal levels based on decision-making process (clinical reasoning) during the different physiotherapy sessions [37, 40, 61]. PAIVM’s were performed either centrally posterior-anterior with directed force toward the spinous process or unilaterally posterior-anterior with force toward the articular pillars [37, 40, 61], with the patient's head pre-positioned according to the PPIVM's. Mobilization grades 1 to 4 were selected according to patient’s tolerance, stiffness subjectively assessed by the examiner, and clinical reasoning [37, 40, 61], i.e., the specific number of sets, repetitions, neck pre-positioning, and mobilization dosage were left to the therapist's interpretation, as would be done in the clinic. The average duration of all sessions was 30 minutes (this duration included ± 15 minutes of mobilization). This included time for pre- and post-treatment assessment and the treatment itself.

At each session, the patient was assessed and asked to rate whether she/he wanted to continue treatment according to her/his improvement. If the patient was satisfied with the reduction in pain intensity, the treatment was stopped. All patients answered a seven-points “globally perceived effect” questionnaire [62]: “Since starting treatment, your current overall condition is: 1=very much improved, 2=much improved, 3=minimally improved, 4=no change, 5= minimally worse and 7=very much worse. After the last mobilizations session, ANSP were immediately assessed for fast neck rotation by the first examiner using the DidRen laser test. The NPRS was also reassessed.

### Statistical analyses

Sample size was calculated for DidRen total time only, as this is the only kinematic variable ever studied in a neck pain population [21] and this kinematic variable was considered the most relevant outcome for the DidRen laser test [22]. To determine the pain effect (difference between ANSP and HCP), the sample size was set at 37 subjects in each group with two-sample t-tests assuming equal variance. To determine the intervention effect (difference between before and after mobilizations), a sample size of 38 subjects was estimated for a paired t-test. For both, the power was 85% with a α at 0.05, the mean difference was determined to be 5.0 seconds, and the standard deviation was determined to be 7.0 for each group.

Intraclass Correlation Coefficient (ICC) calculation was performed using IBM SPSS Statistics-25. All other statistical procedures were performed using SigmaPlot 13 (Systat Software, Inc) with a significance level 0.05.

### Reliability

For each kinematic variable, reliability was assessed between the first (T_1_) and second (T_2_) DidRen laser tests of the HCP group. T_1_ and T_2_ were separated by 20 minutes. Each variable was the result of the average of 5 cycles performed by each healthy control subject during a test.

We used intra-rater reliability with two trials (ICC_3,2_) [63] and with a 2-way random with absolute agreement [64]. ICCs were calculated for each kinematic variable with a 95% confidence interval (95% CI) [65]. Agreements were calculated using the standard errors of measurement (SEM), SEM $= SD\gamma \sqrt{\left(1- R\gamma \right)}$, where *SDγ* = standard deviation of γ = average of results of T_1_ and T_2_, and *Rγ* = ICC between T_1_ and T_2_. Standard errors of measurement were also calculated in percentage relative to the mean, as follow: $RSEM\%=\frac{SEM}{MEAN}$× 100, where *MEAN* = average of all observations of T_1_ and T_2_. The SEM allowed us to calculate the minimum detectable change (MDC) at 95% CI level (*MDC*_*95*_), which was calculated as ${MDC}_{95}=1.96\times \sqrt{2\times SEM}$. MDC was also calculated as a percentage relative to the mean, as follows $RMDC\%=\frac{MDC}{MEAN}$ × 100, where *MEAN* = average of all observations of T_1_ and T_2_.

### Validity

Because male/female equality between the ANSP and HCP groups was not optimal, we examined the influence of gender (gender x groups) on the kinematic variables. We used a two-way ANOVA with *post hoc* Holm-Sidak method for pairwise multiple comparisons when ANOVA indicated a significant interaction.

To assess the effect of painful cervical level (upper/lower) on the kinematic variables, we used a two-way repeated measure ANOVA with *post hoc* Holm-Sidak method for pairwise multiple comparisons was conducted when ANOVA indicated significant interaction.

*“The ANSP patients’ pre-treatment data were compared with the HCP group using ANCOVA (adjusted for age, as the age difference between ANSP patients and HCP groups was large) with post hoc Holm-Sidak method for all pairwise multiple comparisons when ANCOVA indicated a significant interaction.”*

### Intervention

To assess the effect of passive manual therapy interventions, a two-way repeated measure ANOVA was performed with *post hoc* Holm-Sidak method for pairwise multiple comparisons when ANOVA indicated a significant interaction. All data were normally distributed, as confirmed by Shapiro’s test or equal variance test.

Effect sizes (ES) were calculated to provide a more interpretable quantitative description of effect size [66].

## Results

A total of 42 ANSP patients and 54 HCP were screened. Four patients were excluded after the first assessment/treatment because they cancelled their second appointment. Twelve participants were excluded because they were not pain-free (i.e., they experienced pain during passive manual assessment of the cervical spine).

A total of 38 ANSP patients and 42 HCP participated in the study. Table 1 shows the characteristics of the ANSP patients and HCP groups. Table 2 shows the clinical information collected before and after the mobilizations.

**Table 1:** Characteristics of the ANSP and HCP

Global (n=80)	ANSP (n=38)	HCP (n=42)	***P***-values
**Age (years), mean** ± **SD**	46.2 ±16.3	24.3±6.8	<0.001
**Sex n (males/females), (%)**	21 (55%)/17 (45%)	27 (64%)/15 (36%)	0.55
**BMI (kg m**^**-2**^**), mean** ± **SD**	23.5 ±3.2	21.5 ±4.2	0.014
**NDI (100), median [Q1-Q3]**	22 [16-31.5]	0 [0-0]	<0.001
**NPRS, median [Q1-Q3]**	6 [4-7]	0 [0-0]	<0.001
**Active Rotation: Left (SD) - Right (SD)**	59.1°(16.7 °) – 59.7° (13.6 °)	69.7 ° (8.2 °) - 70.4° (6.8°)	0.019 ; 0.014
**TSK median [Q1-Q3]**	38 [31-42]	Not applicable	Not applicable
**Bournemouth, median [Q1-Q3]**	28.5 [15.25-46]	2.1 [0-2.75]	<0.001

**Table 2:** Patients’ clinical information before and after the mobilizations

ANSP (***n***=38)	Before mobilizations	After mobilizations	***P***-values
**NPRS, median [Q1-Q3]**	6 [4-7]	0 [0-1]	<0.001
**Number of patients, with (Upper/Lower) pain location**	(17/21)	Not applicable	Not applicable
**NPRS, due to DidRen laser test [Q1-Q3]**	2 [0-4.75]	0 [0-0]	<0.001
**Upper neck NPRS during DidRen laser test [Q1-Q3]**	1.5 [0-4]	0 [0-0]	<0.001
**Lower neck NPRS during DidRen laser test [Q1-Q3]**	2 [0-4.25]	0 [0-0]	<0.001
**Number of patients who took pain medication (n): NSAID; Paracetamol; Pain Killer**	(7); (4); (1)	(0), (0), (0)	Not applicable
**Number of sessions, mean (SD)**	Not applicable	4.7 (2.3)	Not applicable
**Number of weeks for therapy (SD)**	Not applicable	6 (3.5)	Not applicable

*SD* Standard Deviation, *BMI* Body Mass Index, *Q1* First Quartile, *Q3* Third Quartile, *NSAID* non steroïd anti-inflammatory drug, *NPRS* Numerical Pain Rating Scale

*SD* Standard Deviation, *BMI* Body Mass Index, *Q1* First Quartile, *Q3* Third Quartile, *NDI* Neck Disability Index, *NPRS* Numerical Pain Rating Scale, *TSK* Tampa Scale of Kinesiophobia

### Reliability

The ICC values for the kinematic variables are shown in Table 3. Most ICCs ranged from moderate (0.57 (0.06-0.80)) to excellent (0.96 (0.91-0.98)), with the exception of overshoot (0.08 (-1.02 - 0.57)), time to peak deceleration (0.22 (-0.65 - 0.63)), and time from peak acceleration to end of rotation (0.44 (-0.21- 0.74)). In addition, the 95% CI of the low and moderate ICCs indicate that the results were not homogeneous and showed high variability between subject groups. Results from SEM and MDC are shown in Table 3.

**Table 3:** Results of the ICC, SEM and MDC

Kinematic parameters	ICC (95% CI)	SEM (RSEM%)	MDC (RMDC%)
**ROM test (°)**	0.57 (0.06 - 0.80)	0.58 (-2.14)	1.60 (-5.93)
**Average speed (°s**^**-1**^**)**	0.84 (0.65-0.92)	3.44 (7.17)	9.53 (19.89)
**Peak speed (°s**^**-1**^**)**	0.93 (0.85-0.97)	8.26 (7.39)	22.91 (20.48)
**Peak acceleration (°s**^**-2**^**)**	0.95 (0.8-0.97)	75.65 (12.96)	209.69 (35.93)
**Peak deceleration (°s**^**-2**^**)**	0.96 (0.91-0.98)	71.12 (-8.07)	197.13 (-22.38)
**Time to peak speed (s)**	0.61 (0.17- 0.82)	0.02 (14.86)	0.044 (41.18)
**Time to peak acceleration (s)**	0.81 (0.60 - 0.91)	0.02 (10.05)	0.05 (27.84)
**Time to peak deceleration (s)**	0.22 (-0.65 - 0.63)	0.02 (30.43)	0.05 (84.35)
**Time between peaks acceleration-deceleration (s)**	0.78 (0.53-0.89)	0.01 (-13.01)	0.04 (-36.09)
**Time from peak acceleration to end of rotation (s)**	0.44 (-0.21- 0.74)	0.02 (15.76)	0.06 (43.68)
**Angle at maximum speed (°)**	0.66 (0.27 - 0.84)	0.82 (5.74)	2.27 (15.91)
**Stabilization Time (s)**	0.79 (0.45 - 0.91)	0.14 (8.25)	0.39 (22.87)
**Overshoot (°)**	0.08 (-1.02 - 0.57)	0.22 (35.51)	0.62 (98.44)
**DidRen total time (s)**	0.78 (0.31-0.91)	2.74 (82.40)	7.59 (228.40)

*ICC* Intraclass Correlation Coefficient, *CI* Confidence Interval, *SEM* Standard Error of Measurement, *RSEM%* Relative (%) Standard Error of Measurement expressed as a percentage, *MDC* Minimal Detectable Change, *%RMDC* Relative (%) Minimal Detectable Change expressed as a percentage

Note that ROM test (which is the ROM reached during the DidRen laser test) and active ROM (performed by the patient before the DidRen laser test)

### Validity

There was no significant difference between the genders of ANSP and HCP (see Supplementary Table 1) and no significant difference was found between ANSP with upper versus lower spinal pain localization (see Supplementary Table 2).

Table 4 shows the difference between ANSP (considered in one group: upper and lower spinal pain location) and HCP groups. ANSP were significantly slower in the time between the peaks of acceleration and deceleration (*p*<0.019). This time result was longer than that of SEM (Table 3).

**Table 4:** Results for kinematic variables during the DidRen laser test based on the comparison of ANSP and HCP groups before the intervention, adjusted with the covariate “age” (ANCOVA method)

Variables	Adjusted mean (95% CI)	Difference of the adjusted means of the groups	***P***-values
**ROM test (°)**	26.869 (27.348-26.390)	0.64	0.238
**Average speed (°s**^**-1**^**)**	46.300 (42.992-49.608)	4.666	0.094
**Peak speed (°s**^**-1**^**)**	109.290 (98.563 – 120.017)	12.96	0.169
**Peak acceleration (°s**^**-2**^**)**	566.838 (465.380- 668.295)	116.308	0.173
**Peak deceleration (°s**^**-2**^**)**	-845.499 (-956.433–734.566	153.347	0.101
**Time to peak speed (s)**	0.114 (0.0975 – 0.130)	0.024	0.075
**Time to peak acceleration (s)**	0.176 (0.152 – 0.200)	0.04	0.053
**Time to peak deceleration (s)**	0.0650 (0.0513 – 0.0787)	0.011	0.336
**Time between peaks acceleration-deceleration (s)**	-0.111 (-0.125 –0.0968)	-0.03	0.019 *
**Time from peak acceleration to end of rotation (s)**	0.145 (0.130 – 0.160)	0.011	0.373
**Angle at maximum speed (°)**	14.063 (13.407 – 14.719)	0.60	0.275
**Stabilization Time (s)**	1.853 (1.731 – 1.976)	0.07	0.511
**Overshoot (°)**	0.641 (0.5561 – 722)	0.06	0.335
**DidRen total time (s)**	49.951 (47.107 – 52.794)	4.17	0.082

*ICC* Intraclass Correlation Coefficient, *CI* Confidence Interval, *SEM* Standard Error of Measurement, *RSEM%* Relative (%) Standard Error of Measurement, expressed as a percentage, *MDC* Minimum Detectable Change, *%RMDC* Relative (%) Minimum Detectable Change, expressed as a percentage. Be careful not to confuse this ROM test (which is the ROM reached during the DidRen laser test) with the active ROM test (which is performed by the patient prior to the DidRen laser test)

*P-*values are given for the differences between ANSP and HCP of the adjusted means with the covariate “age”. A bold *P*-Value indicates a significant difference between ANSP and HCP with *p*< 0.05

## Intervention

Table 5 shows that ANSP were significantly faster on several variables after the intervention: peak speed (*p*<0.007), peak acceleration (*p*<0.038) and deceleration (*p*<0.005). They were also faster between peak acceleration and deceleration (*p*<0.002), at stabilizing the laser on target (*p*<0.033) and at performing the DidRen laser test (*p*<0.002). All results were smaller than those of the MDC (Table 3). ES for all kinematic variables ranged from low to medium (Table 5). The largest ES values were observed for DidRen total time, time between peak acceleration and deceleration, average speed, and stabilisation time.

**Table 5:** Results for kinematic variables during DidRen laser test for all ANSP patients according to the effect of intervention and according to “before” and “after” the mobilizations without effect of spinal pain localization

Kinematic Variables	Cervical spine level *vs* before intervention Mean (5%-95% CI)		Cervical spine level *vs* after intervention Mean (5%-95% CI)		Effect Size
	Upper	Lower	Upper	Lower	
ROM Tests (°)	27.104 (22.593-30.946)	27.176 (24.420-29.769)	27.447 (25.726-29.474)	27.235 (25.249-28.738)	-0.14
Average Speed (°s^-1^)	38.985 (18.657-53.482)	41.435 (19.781-59.587)	42.344 (22.548-59.394)	45.084 (31.003-58.831)	0.4
**Peak speed (°s**^**-1**^**)**	**90.502(49.020-144.602)**	**95.156 (45.763-160.791)**	**102.364 (74.783-142.645)**	**100.251 (67.549-146.181)**	0.3
**Peak acceleration (°s**^**-2**^**)**	**398.570 (178.360-862.330)**	**459.258 (126.714-1127.063)**	**481.761 (257.215-912.625)**	**478.915 (251.797-959.518)**	0.2
**Peak deceleration (°s**^**-2**^**)**	**-643.711(-1234.415--236.355)**	**-685.630 (-1308.188--247.149)**	**-758.298 (-1095.220--487.725)**	**-743.563 (-1159.127--420.007)**	-0.3
Time to peak speed (s)	0.149 (0.102-0.318)	0.138 (0.0936-0.331)	0.141 (0.0965-0.274)	0.120 (0.0965-0.201)	-0.3
Time to peak acceleration (s)	0.225(0.157-0.461)	0.218 (0.136-0.529)	0.210 (0.141-0.344)	0.187 (0.143-0.237)	-0.3
Time to peak deceleration (s)	0.0889 (0.0473-0.247)	0.0773 (0.0489-0.239)	0.0879 (0.0462-0.222)	0.0669 (0.0528-0.147)	-0.1
**Time between peaks acceleration-deceleration (s)**	**0.136 (0.0815-0.215)**	**0.141 (0.0791-0.290)**	**0.122 (0.0850-0.153)**	**0.119 (0.0604-0.182)**	0.5
Time from peak acceleration to end of rotation (s)	0.170 (0.0907-0.320)	0.159 (0.0836-0.294)	0.169 (0.0973-0.329)	0.143 (0.0985-0.270)	-0.2
Angle at maximum speed (°)	14.274 (8.176-22.172)	14.390 (10.902-20.214)	14.997 (11.833-20.569)	15.022 (13.130-23.262)	0.3
**Stabilisation Time (s)**	**1.908 (1.163-2.536)**	**1.996 (1.567-2.602)**	**1.834 (1.501-2.397)**	**1.830 (1.178-2.486)**	-0.4
Overshoot	0.679 (0.267-1.255)	0.737 (0.323-1.325)	0.610 (0.282-0.894)	0.682 (0.339-1.475)	0.2
**DidRen time**	**54.36 (41.702-81.389 )**	**53.00 (45.657-79.465)**	**55.36 (41.531-67.405)**	**50.35 (36.755-65.883)**	-0.5

*Vs* means versus. Variables and results and in bold denote significant differences observed after intervention (without the effect of spinal pain localization (see Supplementary Table 2)) with *p*< 0.05. Results of the intervention are reported by cervical levels (Upper and Lower) because patients were assigned to these two different groups before the intervention

The average duration of “treatment” was 15 minutes. The mean number of treatment sessions was 4.7 (±2.3) and the mean number of treatment per week was 3.8 (±2.6). The NPRS averaged 5.6 (± 1.7) at baseline and 0.5 (± 0.8) at the end of intervention.

For “global perceived effect”, 35 patients were “very much improved” and “much improved”. Three patients were “slightly worse” and averaged 8 treatment sessions”.

## Discussion

The results of this study confirm moderate to good reliability of most outcome variables of the DidRen laser test when examined with healthy subjects [21]. This study showed that a kinematic variable (time between peaks of acceleration and deceleration) was significantly altered during fast head axial rotations in patients with acute-subacute non-specific neck pain compared with healthy control subjects. Furthermore, our study showed improvement in some kinematic variables after passive manual therapy intervention along with pain reduction.

### Reliability

Moderate to excellent intra-individual reliability was observed for all kinematic variables except overshoot, time to peak deceleration, and time to peak acceleration to end of rotation. For average and peak speed, our ICC values in healthy control subjects concur with those of Sarig Bahat (2016), who determined the inter-tester reliability of similar kinematic measurements using a virtual reality system in asymptomatic subjects [67]. In line with our observations, they also reported moderate reliability for rotation velocity and good reliability for peak velocity [67].

The lower reliability of the results for the overshoot, time to peak deceleration, and time to peak acceleration to end of rotation variables can be explained in part by the fact that these kinematic variables have greater variability among individuals. In the ANSP patient group, we demonstrated before and after the intervention that all significant results were below the MDC. Researchers and clinicians should look for ways to challenge the sensorimotor control system to a greater extent. The DidRen was able to distinguish ANSP patients from HCP, but not enough to be clinically meaningful.

### Validity

Given the observed differences in neck sensorimotor control performance between ANSP patients and HCP, further studies are warranted to investigate the internal and external validity of these findings. Our results confirm previous observations of kinematic behavior in patients with acute and chronic neck pain [18, 43]. Our hypothesis that poor sensorimotor control during head rotation would result in significantly impaired kinematic variables, such as prolonged times in performing a series of accurate and constraining neck movements, was supported. We are aware that the kinematic results of our test are directly related to the establishment of sensorimotor control of function, which also depends on the level of tone that allows eliminating the degrees of freedom (variability of movement) [68]. Therefore, the significant difference obtained in the time between acceleration and deceleration during the typical DidRen “target-to-target” movement in a position-velocity plane is relevant. Indeed, the nervous system is always confronted with problems of selection among an infinite number of possibilities when adapting the movement to the required task, [69]. Profeta et al. (2018) stated that in order to limit the degrees of freedom and ensure the coherence of movements, proprioceptive relays in the form of feedforward and feedback are essential for optimal performance [70, 71]. Therefore, the reduction in time observed in patients with variable time between acceleration and deceleration peaks is probably related to an impairment of cervical feedback and anticipation, i.e. sensorimotor control. This indicates that patients manage their dynamic degrees of freedom with less adaptability during task execution. However, our results contrast with de Zoete et al. who found no differences in cervical spine sensorimotor control between subjects with chronic idiopathic neck pain and asymptomatic control subjects [72]. Only the “Fly”, which measures accuracy following tracking a moving target but does not include a speed component, could be compared to our test.

As for our significant result, we can conclude that pain could be responsible for the observed differences between patients and healthy control subjects. Three hypotheses could explain such observed differences between patients and healthy control subjects including (1) enhanced sensorimotor control of the neck via different sensorimotor channels acting together (i.e., neck, vestibular and ocular reflexes). During the DidRen laser test, proprioception, vestibular, and visuomotor control simultaneously contribute to the coordination of the head and eye movement control to ensure performance [22]. Thus, a non-specific test that uses different sensorimotor channels, such as the DidRen laser test, may produce results that can be associated with pain; (2) the principle of the DidRen laser test is in line with Panjabi’s theory and Riemann’s definition of vertebral stability. The authors state that the different structures that provide spinal stability can be divided into interdependent systems: the passive, the active and the nervous systems [26, 73, 74]. According to Panjabi, the passive system consists of the ligaments and joint capsule. The active and neural systems are the dynamic parts that result from neuromotor control trough feedforward and feedback from the spinal muscles that pass through the joint. Under abnormal conditions, such as after trauma or a degenerative process, or even pain, the interaction of the passive or/and active or/and neural systems can disrupt and affect the stabilization processes of the neck. Neck stabilization is more important in the neutral zone (i.e., the zone of high flexibility or laxity), which is from 0° to 29.6° for unilateral axial rotation on C_1_-C_2_ [26]. Thus, with a mean head rotation amplitude of ±27° achieved by patients when performing the DidRen laser test, we can assume that our test is more likely to affect the neutral zone, which could be disturbed by acute-subacute neck pain; and (3) sensorimotor performance could be integrated into the decision-making framework: “Reach the targets as fast as possible”. Therefore, participants had to adjust their speed during the dynamic phase and their accuracy during the stabilization phase during the axial rotation [75]. The speed-accuracy trade-off could be considered a “signature” of the decision-making process [76]. This varies depending on which movement behavior is the focus: e.g. accuracy or speed [75] during a target task that includes the amplitude of the movement, the size, and the position of the target [75, 77, 78]. Assessing the speed-accuracy trade-off was not the goal of our study, but interestingly, this trade-off is fundamental assumption that could explain our results. Indeed, we showed that ANSP patients become slower as accurate as HCP (no significant difference in overshoot). Moreover, our results showed that the analysis of the overshoot variable is apparently representative of the quality of the sensorimotor status of the neck and fits the speed accuracy trade-off in the neutral zone [19, 23].

We were unable to confirm our hypothesis that sensorimotor dysfunction would play a greater role in participants with acute-subacute neck pain originating from the upper cervical spinal levels (C_0_ to C_2_), as has been observed in patients with traumatic neck pain [8, 79]. This could be explained by the insufficient sample size (in the groups with upper (*n*=17) and lower (*n*=21) cervical spine pain). Nevertheless, with a sample size comparable to ours (upper (*n*=21) and lower (n=15)), Treleaven et al. showed comparable results with the joint position error test. A second reason could also be that the “axial rotation test” described by Satput et al (2019) [80] is not accurate enough and that some patients classified as having upper cervical spine pain were not.

### Intervention

To assess pain-related changes in sensorimotor control, we chose passive manual therapy mobilizations, which are known to reduce neck pain [40]. Our results that passive manual mobilizations improved sensorimotor kinematic variables are consistent with other studies that showed that more accurate proprioceptive input processing is enhanced by reducing pain effects [81, 82]. For significant kinematic variables between pre- and post-intervention, ES ranges from low to moderate. These effects were obtained after ± 6 weeks of passive mobilizations and can be compared with the ES obtained by Meisingset et al. after 8 weeks of physiotherapy, which included a wide range of modalities assessed with the Fly test [33].

### Clinical implications

Clinicians could gain relevant insights into sensorimotor control by assessing the rotational movements of the head-neck complex as part of their assessment of acute-subacute patients with neck pain. This highlights the interest in the diagnostic process to distinguish patients with acute-subacute non-specific neck pain patients from asymptomatic individuals to determine a cut-off point that may be clinically relevant.

In view of our findings, it would be useful to further investigate the various aspects of cervical rotation acceleration and deceleration for rehabilitation of patients with neck pain”.

### Limitations and strengths

The results of the current study should be viewed in light of several methodological limitations. First, we calculated the reliability only in healthy subjects because, according to the results of Roijezon et al (2010) [13], we wanted a good reference value for this sensorimotor control test that was not influenced/altered by neck pain. In this article, the SEM of the kinematic variables calculated in the control subjects was always higher than in the patients with neck pain, with the exception of the conjunctive movements. However, this might not be the case for the outcome variables and in our healthy subjects and secondly, it might undermine the effect generated by our intervention.

Second, the age did not match between the group of patients and the healthy control group. Twelve control participants were in fact excluded because they reported pain during the clinical examination (PAIVM’s). These were mainly elderly individuals. Indeed, the causal inference of a control group may be affected by various (as yet unknown) clinical biases. Since the prevalence of neck pain increases with age and older patients are likely to have residual effects on neck motor control performance due to previous pain, it is possible that factors other than those studied are responsible for the observed associations [83]. To account for this possible confounder, age was included in the ANCOVA test.

Third, due to the sample size, this study could be considered a pilot trial. Future studies with more diverse ethnic background could be conducted to increase the external validity of the findings. In addition, a relatively large sample could have been more representative. Fourth, recruitment bias cannot be excluded. Namely, the patients were referred to an experienced orthopaedic manual physiotherapist who is known as specialist in neck care. Therefore, a multicenter study with different physiotherapists would have been methodologically more appropriate.

Finally, the pain reduction could also be related to the learning effect of the test. However, Bootsma et al. [84] have shown that task difficulty affects motor performance, but not learning. Therefore, it can be assumed that the learning effect is small. This should be studied in future experiments. Although this prospective study provides interesting results, they should be confirmed in randomized comparison trials with longer follow-ups.

The strength of this study is that for the first time, a sensorimotor assessment of patients with acute-subacute non-specific neck pain was performed with a pragmatic intervention and a follow-up test immediately after the completion of the intervention.

In order to have a more sophisticated reference measure, we calculated reliability using healthy subjects, without knowing whether these results would have translated for our patients.

## Conclusions

The moderate to good reliability of the DidRen laser test allowed us to demonstrate the change in a kinematic variable (reduction in time between acceleration and deceleration peaks) in a sample of patients suffering from acute-subacute neck pain compared to healthy participants. Contrary to our original hypothesis, there were no differences in sensorimotor control when we compared patients with upper versus lower cervical spine pain levels. We found that neck pain decreased after passive manual therapy mobilizations, resulting in statistically but not clinically significant effects on several kinematic variables. These results suggest that sensorimotor changes may occur rapidly after pain subsides. The present study is of importance because, to our knowledge, previous studies of sensorimotor control have included mainly chronic non-specific neck pain populations, and this is the first study to examine sensorimotor control of the neck only in acute-subacute non-specific neck pain patients.

## Supplementary Information

**Additional file 1: Figure S1.** Example of C_0_-C_2_ axial rotation test to the left (posterior view). The patient was examined in a standardized sitting position with the neck in neutral position. The assessor passively rotated the patient’s head to the left with C_2_ stabilized and the assessor’s thumb and index fingers to isolate superior cervical levels from below.

**Additional file 2: Figure S2**. Examples of PPIVM’s (Passive Physiological Intervertebral Movement’s) in Lateral flexion. Lateral flexion to the left on C_1_ (A) and C_5_ (B) with hand placement (“patient” in supine with the “head” beyond the end of the couch). With both hands, the assessor gave support under the occiput. The assessor applied the thumbs directed laterally to the articular pillars from the upper cervical region C_1_ (A) to the lower region C_5_ (B) on each side.

**Additional file 3: Figure S**3**.** Examples of PAIVM’s (Passive Accessory Intervertebral Movement’s). Right unilateral on C_2_ (A) and C_6_ (B) with hand placement (“patient” in prone with the “head” on the right side of each picture). The assessor applied his thumb directed posterior-anterior force to the articular pillars from the upper cervical region C_0-1_, C_1-2_ and C_2-3_ to the lower region C_6-7_ on each side.

**Additional file 4: Supplemental Table 1.** Effect of the gender (gender x groups) on the kinematic variables. two-way ANOVA with *post hoc* Holm-Sidak method for pairwise multiple comparisons when ANOVA indicated significant interaction.

**Additional file 5: Supplemental Table 2.** Results of a two-way repeated measure ANOVA with *post hoc* Holm-Sidak method for pairwise multiple comparisons was conducted when ANOVA indicated significant interaction to calculate *P*-Values for kinematic variables during DidRen laser test for all ANSP patients according to the spinal pain location (upper/lower spine levels) and according of the effect of intervention.

**Additional file 6.**

**Additional file 7.**

## Data Availability

All data generated or analyzed during this study are included in this published article [and its supplementary information files].
