# Supplementary material for: Sensorimotor performance in acute-subacute non-specific neck pain: a non-randomized prospective clinical trial with intervention
Source: BMC Musculoskelet Disord. 2021 Dec 4;22:1017. doi: 10.1186/s12891-021-04876-4 (PMC8645120; doi:10.1186/s12891-021-04876-4)
Supplement: Supplementary file 1 — Additional file 1: Figure S1. Example of C0-C2 axial rotation test to the left (posterior view). The patient was examined in a standardized sitting position with the neck in neutral position. The assessor passively rotated the patient’s head to the left with C2 stabilized and the assessor’s thumb and index fingers to isolate superior cervical levels from below. [file 12891_2021_4876_MOESM1_ESM.docx]

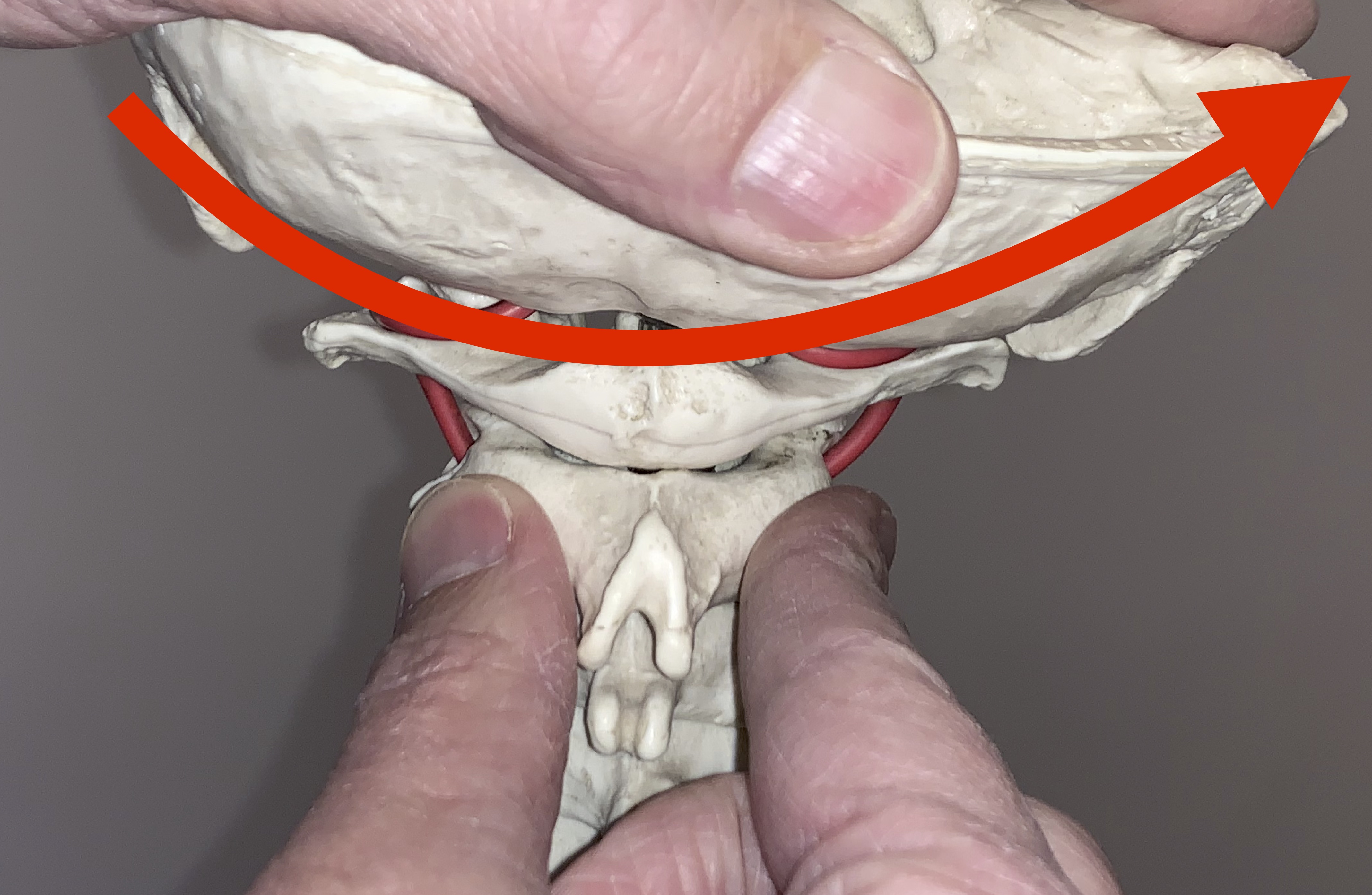


Fig 1. **Example of C_0_-C_2_ axial rotation test to the left (posterior view).** The patient was examined in a standardized sitting position with the neck in neutral position. The assessor passively rotated the patient’s head to the left with C_2_ stabilized and the assessor’s thumb and index fingers to isolate superior cervical levels from below.
