# Supplementary material for: Sensorimotor performance in acute-subacute non-specific neck pain: a non-randomized prospective clinical trial with intervention
Source: BMC Musculoskelet Disord. 2021 Dec 4;22:1017. doi: 10.1186/s12891-021-04876-4 (PMC8645120; doi:10.1186/s12891-021-04876-4)
Supplement: Supplementary file 2 — Additional file 2: Figure S2. Examples of PPIVM’s (Passive Physiological Intervertebral Movement’s) in Lateral flexion. Lateral flexion to the left on C1 (A) and C5 (B) with hand placement (“patient” in supine with the “head” beyond the end of the couch). With both hands, the assessor gave support under the occiput. The assessor applied the thumbs directed laterally to the articular pillars from the upper cervical region C1 (A) to the lower region C5 (B) on each side. [file 12891_2021_4876_MOESM2_ESM.docx]

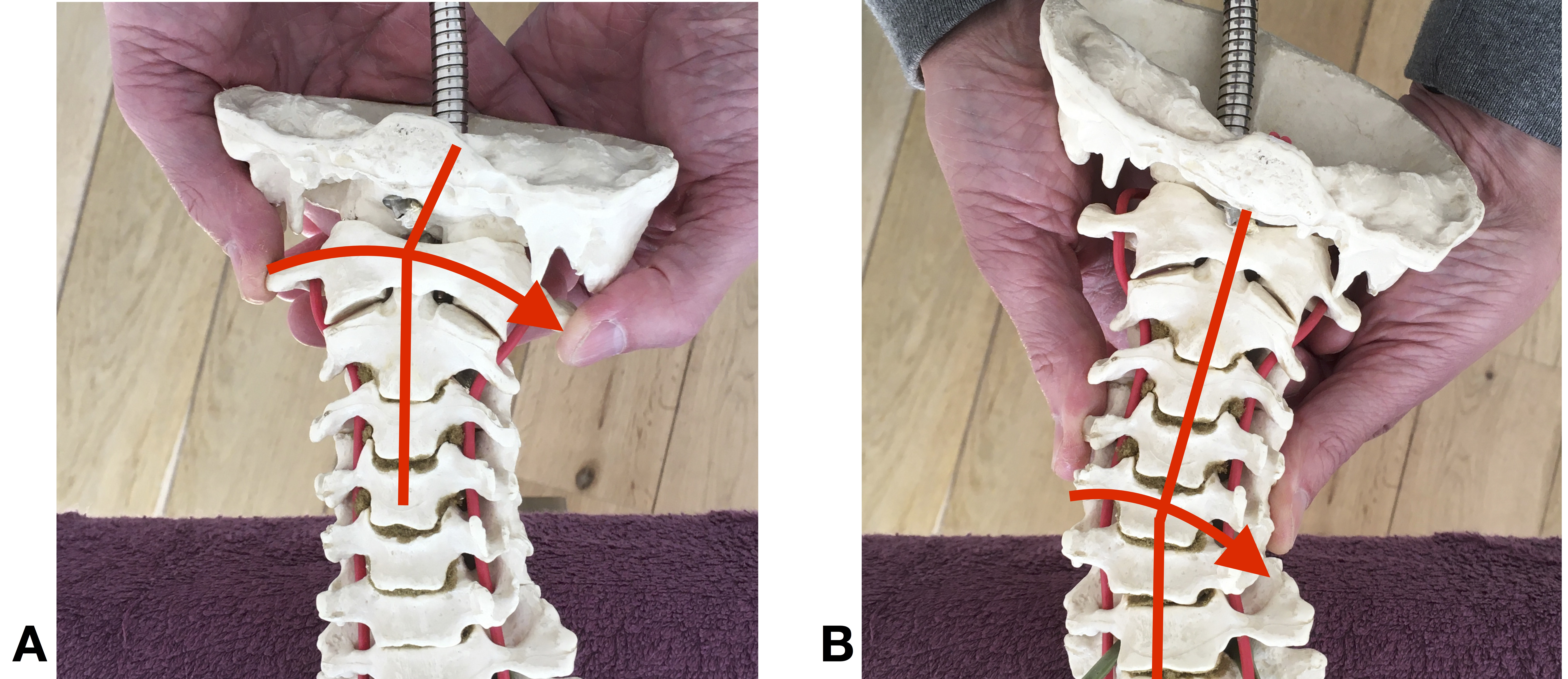


Fig 2. **Examples of PPIVM’s (Passive Physiological Intervertebral Movement’s) in Lateral flexion.** Lateral flexion to the left on C_1_ (A) and C_5_ (B) with hand placement (“patient” in supine with the “head” beyond the end of the couch). With both hands, the assessor gave support under the occiput. The assessor applied the thumbs directed laterally to the articular pillars from the upper cervical region C_1_ (A) to the lower region C_5_ (B) on each side.
