# Supplementary material for: Sensorimotor performance in acute-subacute non-specific neck pain: a non-randomized prospective clinical trial with intervention
Source: BMC Musculoskelet Disord. 2021 Dec 4;22:1017. doi: 10.1186/s12891-021-04876-4 (PMC8645120; doi:10.1186/s12891-021-04876-4)
Supplement: Supplementary file 3 — Additional file 3: Figure S3. Examples of PAIVM’s (Passive Accessory Intervertebral Movement’s). Right unilateral on C2 (A) and C6 (B) with hand placement (“patient” in prone with the “head” on the right side of each picture). The assessor applied his thumb directed posterior-anterior force to the articular pillars from the upper cervical region C0-1, C1-2 and C2-3 to the lower region C6-7 on each side. [file 12891_2021_4876_MOESM3_ESM.docx]

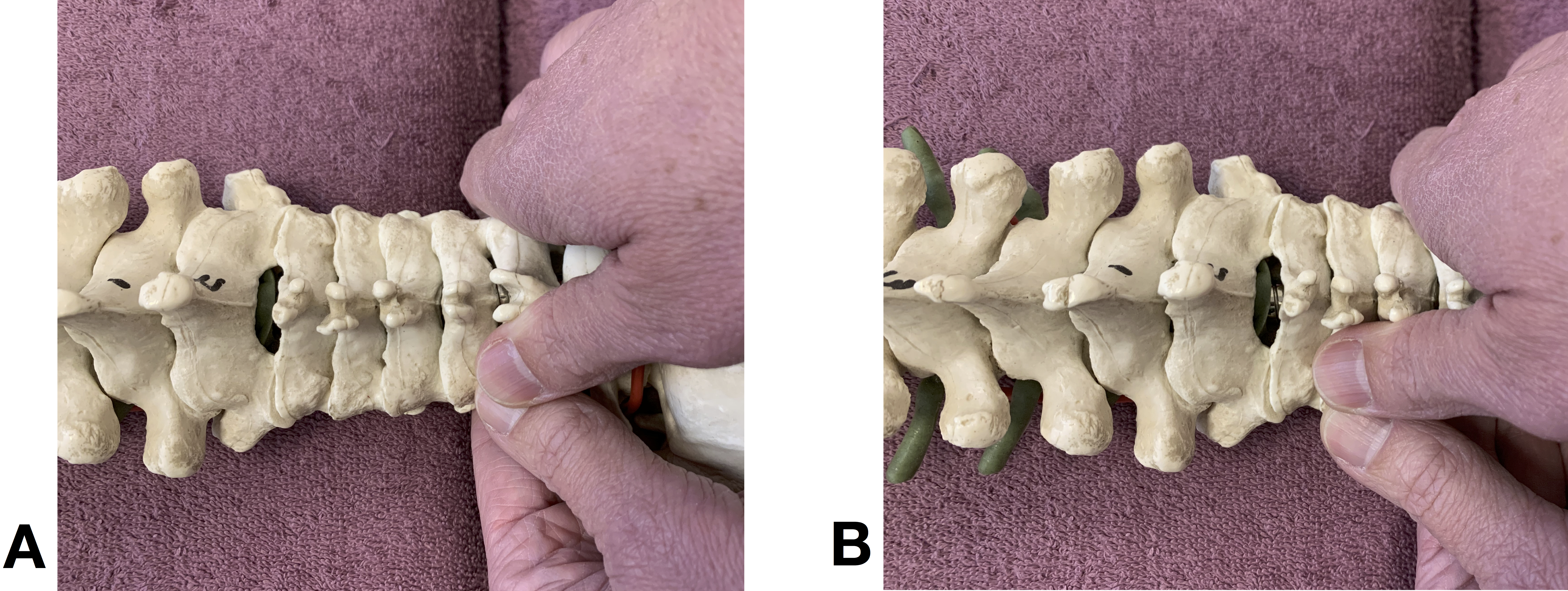


Fig 3. Examples of PAIVM’s (Passive Accessory Intervertebral Movement’s). Right unilateral on C_2_ (A) and C_6_ (B) with hand placement (“patient” in prone with the “head” on the right side of each picture). The assessor applied his thumb directed posterior-anterior force to the articular pillars from the upper cervical region C_0-1_, C_1-2_ and C_2-3_ to the lower region C_6-7_ on each side.
