# Supplementary material for: Sensorimotor performance in acute-subacute non-specific neck pain: a non-randomized prospective clinical trial with intervention
Source: BMC Musculoskelet Disord. 2021 Dec 4;22:1017. doi: 10.1186/s12891-021-04876-4 (PMC8645120; doi:10.1186/s12891-021-04876-4)
Supplement: Supplementary file 4 — Additional file 4: Supplemental Table 1. Effect of the gender (gender x groups) on the kinematic variables. two-way ANOVA with post hoc Holm-Sidak method for pairwise multiple comparisons when ANOVA indicated significant interaction. [file 12891_2021_4876_MOESM4_ESM.docx]

Supplemental Table 1: Effect of the gender (gender x groups) on the kinematic variables. two-way ANOVA with *post hoc* Holm-Sidak method for pairwise multiple comparisons when ANOVA indicated significant interaction.

| Kinematic  parameters | 2W ANOVA | *p*<0.05 |
| --- | --- | --- |
| ROM Test (°) | Gender | 0.026 |
|  | State | 0.793 |
|  | Gender x group | 0.341 |
| Average speed (°s^-1^) | Gender | 0.038 |
|  | State | 0.003 |
|  | Gender x group | 0.498 |
| Peak speed (°s^-1^) | Gender | 0.011 |
|  | State | 0.015 |
|  | Gender x group | 0.861 |
| Peak acceleration  (°s^-2^) | Gender | 0.017 |
|  | State | 0.034 |
|  | Gender x group | 0.819 |
| Peak deceleration (°s^-2^) | Gender | 0.017 |
|  | State | 0.010 |
|  | Gender x group | 0.695 |
| Time to peak speed (s) | Gender | 0.830 |
|  | State | 0.002 |
|  | Gende Gender x group | 0.230 |
| Time to peak acceleration (s) | Gender | 0.763 |
|  | State | 0.003 |
|  | Gender x group | 0.352 |
| Time to peak deceleration (s) | Gender | 0.367 |
|  | State | 0.015 |
|  | Gender x group | 0.166 |
| Time between peaks acceleration-deceleration (s) | Gender | 0.151 |
|  | State | 0.006 |
|  | Ge Gender x group | 0.796 |
| Time from peak acceleration to end of rotation (s) | Gender | 0.519 |
|  | State | 0.016 |
|  | Gender x group | 0.490 |
| Angle at maximum speed (°) | Gender | 0.961 |
|  | State | 0.885 |
|  | Gend Gender x group | 0.630 |
| Stabilisation Time (s) | Gender | 0.401 |
|  | State | 0.084 |
|  | Gender x group | 0.643 |
| Overshoot | Gender | 0.043 |
|  | State | 0.163 |
|  | Gender x group | 0.495 |
| DidRen time | Gender | 0.171 |
|  | State | 0.004 |
|  | Gender x group | 0.609 |

*P*-values *p*< 0.05 mean significant differences.
