# Supplementary material for: Sensorimotor performance in acute-subacute non-specific neck pain: a non-randomized prospective clinical trial with intervention
Source: BMC Musculoskelet Disord. 2021 Dec 4;22:1017. doi: 10.1186/s12891-021-04876-4 (PMC8645120; doi:10.1186/s12891-021-04876-4)
Supplement: Supplementary file 5 — Additional file 5: Supplemental Table 2. Results of a two-way repeated measure ANOVA with post hoc Holm-Sidak method for pairwise multiple comparisons was conducted when ANOVA indicated significant interaction to calculate P-Values for kinematic variables during DidRen laser test for all ANSP patients according to the spinal pain location (upper/lower spine levels) and according of the effect of intervention. [file 12891_2021_4876_MOESM5_ESM.docx]

Supplemental Table 2. Results of a two-way repeated measure ANOVA with *post hoc* Holm-Sidak method for pairwise multiple comparisons was conducted when ANOVA indicated significant interaction to calculate P-Values for kinematic variables during DidRen laser test for all ANSP patients according to the spinal pain location (upper/lower spine levels) and according of the effect of intervention.

| **Kinematic**  **Variables** | ***p*<0.05** | | |
| --- | --- | --- | --- |
|  | Cx Levels | B/A | Cx Levels *vs* B/A |
| ROM Tests (°) | 0.839 | 0.460 | 0.602 |
| Average Speed (°s^-1^) | 0.341 | 0.052 | 0.934 |
| Peak speed (°s^-1^) | 0.870 | **0.007** | 0.260 |
| Peak acceleration  (°s^-2^) | 0.670 | **0.038** | 0.191 |
| Peak deceleration (°s^-2^) | 0.861 | **0.005** | 0.338 |
| Time to peak speed (s) | 0.229 | 0.265 | 0.640 |
| Time to peak acceleration (s) | 0.433 | 0.105 | 0.569 |
| Time to peak deceleration (s) | 0.178 | 0.596 | 0.658 |
| Time between peaks acceleration-deceleration (s) | 0.923 | **0.002** | 0.500 |
| Time from peak acceleration to end of rotation (s) | 0.213 | 0.417 | 0.481 |
| Angle at maximum speed (°) | 0.899 | 0.176 | 0.926 |
| Stabilisation Time (s) | 0.657 | **0.033** | 0.398 |
| Overshoot | 0.259 | 0.274 | 0.896 |
| DidRen time | 0.957 | **0.002** | 0.334 |

Cx Levels =cervical upper/lower spine levels. B/A= Before/After intervention. Vs mean versus. In bold mean significant differences with P-values < 0.05.
