# Supplementary material for: Sensorimotor performance in acute-subacute non-specific neck pain: a non-randomized prospective clinical trial with intervention
Source: BMC Musculoskelet Disord. 2021 Dec 4;22:1017. doi: 10.1186/s12891-021-04876-4 (PMC8645120; doi:10.1186/s12891-021-04876-4)
Supplement: Supplementary file 7 — Additional file 7. [file 12891_2021_4876_MOESM7_ESM.pdf]

Dossier participant contrôle

Données personnelles:

N° de dossier :

Sexe:

Age:

N° de téléphone:

Critères d'exclusion:

Plaintes de nuque durant la dernières année:	oui	non
Symptômes irradiant dans l'épaule ou le bras:	oui	non
Maux de tête:	oui	non
Histoire de traumatisme à la nuque:	oui	non
Traité(e) pour trouble de la colonne vertébrale		
(Conservateur ou Chirurgie):	oui	non
Vertiges	oui	non

Questionnaires et échelles:

- *NPRS:*
- *NDI:*
- *The French Version of the Bournemouth Questionnaire:*

NPRS

Echelle numérique (EN)												
Pas de Douleur	0	1	2	3	4	5	6	7	8	9	10	Douleur maximale imaginable

Le consentement éclairé

"*Assessment of the short-term influence of a cervical pragmatic treatment on kinematic strategies during a fast-head rotation task standardized with the "DidRen Laser" device.*"

Participant

Je déclare avoir lu et compris la lettre d'information qui m'a été donnée et avoir été informé(e) sur la nature de l'étude, son but, sa durée, les effets secondaires éventuels et ce que l'on attend de moi. J'ai pris connaissance du document d'information et des annexes à ce document.

J'ai eu suffisamment de temps pour y réfléchir et en parler avec une personne de mon choix (médecin généraliste, parent).

J'ai eu l'occasion de poser toutes les questions qui me sont venues à l'esprit et j'ai obtenu une réponse favorable à mes questions.

J'ai compris que des données me concernant seront récoltées pendant toute ma participation à cette étude et que l'investigateur principal et le promoteur de l'étude se portent garant de la confidentialité de ces données.

Je consens au traitement de mes données personnelles selon les modalités décrites dans la rubrique traitant de garanties de confidentialité . Je donne également mon accord au transfert et au traitement de ces données dans d'autres pays que la Belgique.

J'ai donné librement mon consentement pour participer à cette étude.

J'accepte

que les données de recherche récoltées pour les objectifs de la présente étude puissent être traitées ultérieurement pour autant que ce traitement soit limité au contexte de la présente étude.

« J'ai reçu une copie de l'information au participant et du consentement éclairé ».

Nom, prénom, date et signature du volontaire ou de son tuteur légal.

NDI

Lisez bien les instructions pour chacune des questions, et répondez à toutes les questions. Merci de votre compréhension.

NOM : .... Date : ..../......../........ Heure :........ h ....

ÉCHELLE D'INCAPACITÉ CERVICALE

Ce questionnaire a été établi afin de permettre à votre médecin d'apprécier le retentissement de vos douleurs cervicales sur votre vie au quotidien. Veuillez répondre à toutes les questions en ne cochant que **LA** case qui vous correspond le mieux.

Bien que 2 réponses dans une même rubrique puissent vous correspondre, nous vous remercions de ne cocher qu'une seule case, celle qui se rapporte plus précisément à votre cas.

RUBRIQUE 1 : intensité des douleurs cervicales

- ☐ Je n'ai pas de douleur en ce moment.
- ☐ La douleur est très légère en ce moment.
- ☐ La douleur est moyenne en ce moment.
- ☐ La douleur est très intense en ce moment.
- ☐ La douleur est assez intense en ce moment.
- ☐ La douleur est la pire que je puisse imaginer en ce moment.

RUBRIQUE 2 : soins personnels (se laver, s'habiller, etc.)

- ☐ Je peux prendre soin de moi normalement, sans entraîner plus de douleurs que d'ordinaire.
- ☐ Je peux prendre soin de moi normalement, mais cela provoque plus de douleurs que d'ordinaire.
- ☐ M'occuper de moi est douloureux, et je le fais lentement et avec précaution.
- ☐ J'ai besoin d'aide mais je me débrouille pour la plupart de mes soins personnels.
- ☐ J'ai besoin d'une aide quotidienne pour la plupart de mes soins personnels.
- ☐ Je ne peux pas m'habiller, je me lave avec difficulté, et je reste au lit.

RUBRIQUE 3 : soulever des charges

- ☐ Je peux soulever des charges lourdes, sans plus de douleurs que d'ordinaire.
- ☐ Je peux soulever des charges lourdes, mais cela provoque plus de douleurs que d'ordinaire.
- ☐ Les douleurs cervicales m'empêchent de soulever des charges lourdes du sol, mais je peux y arriver si elles sont placées commodément, par exemple sur une table.
- ☐ Les douleurs cervicales m'empêchent de soulever des charges lourdes, mais je peux soulever des charges.
- ☐ Je ne peux soulever que de très légères charges, moyennes ou légères, si elles sont posées commodément.
- ☐ Je ne peux rien soulever ou porter du tout.

RUBRIQUE 4 : lecture

- ☐ Je peux lire autant que je le veux, sans douleur cervicale.
- ☐ Je peux lire autant que je le veux, avec de légères douleurs cervicales.
- ☐ Je peux lire autant que je le veux, avec des douleurs cervicales modérées.
- ☐ Je ne peux pas lire autant que je le veux à cause de douleurs cervicales modérées.
- ☐ Je peux à peine lire à cause de douleurs cervicales intenses.
- ☐ Je ne peux pas lire du tout à cause de mes douleurs cervicales.

RUBRIQUE 5 : maux de tête

- ☐ Je n'ai pas du tout de maux de tête.
- ☐ J'ai des maux de tête légers et peu fréquents.
- ☐ J'ai des maux de tête modérés et peu fréquents.
- ☐ J'ai des maux de tête modérés et fréquents.
- ☐ J'ai des maux de tête intenses et fréquents.
- ☐ J'ai presque tout le temps des maux de tête.

RUBRIQUE 6 : concentration

- ☐ Je peux me concentrer complètement sans difficulté quand je le veux.
- ☐ Je peux me concentrer complètement avec de légères difficultés quand je le veux.
- ☐ Il m'est relativement difficile de me concentrer quand je le veux.
- ☐ J'ai beaucoup de difficultés à me concentrer quand je le veux.
- ☐ J'ai d'énormes difficultés à me concentrer quand je le veux.
- ☐ Je n'arrive pas du tout à me concentrer.

RUBRIQUE 7 : travail (professionnel ou personnel)

- ☐ Je peux travailler autant que je le veux.
- ☐ Je ne peux faire que mon travail courant, mais rien de plus.
- ☐ Je peux faire la plus grande partie de mon travail courant, mais rien de plus.
- ☐ Je ne peux pas faire mon travail courant.
- ☐ Je peux à peine travailler.
- ☐ Je ne peux pas travailler du tout.

RUBRIQUE 8 : conduite

- ☐ Je peux conduire ma voiture sans aucune douleur cervicale.
- ☐ Je peux conduire ma voiture autant que je le veux, avec de légères douleurs cervicales.
- ☐ Je peux conduire ma voiture autant que je le veux, avec des douleurs cervicales modérées.
- ☐ Je ne peux pas conduire ma voiture autant que je le veux, en raison de douleurs cervicales modérées.
- ☐ Je peux à peine conduire en raison de douleurs cervicales intenses.
- ☐ Je ne peux pas du tout conduire ma voiture à cause des douleurs cervicales.

RUBRIQUE 9 : sommeil (avec ou sans prise médicamenteuse)

- ☐ Mon sommeil n'est pas perturbé.
- ☐ Mon sommeil est à peine perturbé (moins d'une heure sans dormir).
- ☐ Mon sommeil est un peu perturbé (1-2 h sans dormir).
- ☐ Mon sommeil est modérément perturbé (2-3 h sans dormir).
- ☐ Mon sommeil est très perturbé (3-5 h sans dormir).
- ☐ Mon sommeil est complètement perturbé (5-7 h sans dormir).

RUBRIQUE 10 : loisirs (cuisine, sports, activités manuelles, etc.)

- ☐ Je peux participer à toutes mes activités de loisirs sans aucune douleur cervicale.
- ☐ Je peux participer à toutes mes activités de loisirs, avec quelques douleurs cervicales.
- ☐ Je peux participer à la plupart de mes activités habituelles de loisirs, mais pas à toutes à cause de mes douleurs cervicales.
- ☐ Je ne peux participer qu'à quelques-unes de mes activités de loisirs habituelles à cause de mes douleurs cervicales.
- ☐ Je peux à peine participer à des activités de loisirs à cause de mes douleurs cervicales.
- ☐ Je ne peux participer à aucune activité de loisir à cause de mes douleurs cervicales.

Merci de vérifier que vous avez répondu à toutes les questions.

The French Version of the Bournemouth Questionnaire:

Appendix 1 *French adaptation of Bournemouth Questionnaire*

Overall Dimensions for Cervical Bournemouth Questionnaire

Les questions suivantes ont pour objectif de décrire votre douleur cervicale et comment celle-ci vous affecte. Veuillez, s'il vous plaît, répondre à TOUTES les questions en encerclant LE chiffre pour CHAQUE question qui décrit le mieux comment vous vous sentez :

1. Au cours de la dernière semaine, en moyenne, comment évaluez-vous votre douleur cervicale?

Aucune douleur — Pire douleur imaginable

0 1 2 3 4 5 6 7 8 9 10

2. Au cours de la dernière semaine, comment votre douleur cervicale a-t-elle affecté vos activités quotidiennes (effectuer les tâches ménagères, vous laver, vous habiller, lever des charges, lire, conduire)?

Aucun effet — Incapable d'effectuer ces activités

0 1 2 3 4 5 6 7 8 9 10

3. Au cours de la dernière semaine, comment votre douleur cervicale a-t-elle affecté votre habileté à prendre part à des activités récréatives, sociales et familiales?

Aucun effet — Incapable d'effectuer ces activités

0 1 2 3 4 5 6 7 8 9 10

4. Au cours de la dernière semaine, quel a été votre niveau d'anxiété (tension, nervosité, irritabilité, difficulté à se concentrer ou à relaxer)?

Aucune anxiété — Extrêmement anxieux

0 1 2 3 4 5 6 7 8 9 10

5. Au cours de la dernière semaine, avez-vous eu le sentiment d'être déprimé (avoir le cafard, se sentir triste, se sentir déprimé, être pessimiste, se sentir malheureux)?

Aucun sentiment d'être déprimé — Extrêmement déprimé

0 1 2 3 4 5 6 7 8 9 10

6. Au cours de la dernière semaine, comment votre travail (à l'intérieur ou à l'extérieur de la maison) a-t-il affecté (ou affecterait-il) votre douleur cervicale?

Aucune aggravation — Aggravation très importante

0 1 2 3 4 5 6 7 8 9 10

7. Au cours de la dernière semaine, comment avez-vous été capable de contrôler (diminuer/aider) votre douleur cervicale par vous-même?

Contrôle complet — Aucun contrôle

0 1 2 3 4 5 6 7 8 9 10

Examen Clinique

- DidRen Laser Test (x 2/20')

  Douleur après le test (0-10):

- Rotation Active Cinématique

  Droite :
  Gauche :
  Douleur pendant le test DidRen (0-10) :

- Examen vertebral manuel

  - PC2R (raideur/douleur)

  - PAIVM's (raideur/douleur)

  - PPIVM's (raideur/douleur)
